# A Versatile Platform for Designing and Fabricating Multi-Material Perfusable 3D Microvasculatures

**DOI:** 10.3390/mi16060691

**Published:** 2025-06-08

**Authors:** Nathaniel Harris, Charles Miller, Min Zou

**Affiliations:** 1 Department of Mechanical Engineering, University of Arkansas, Fayetteville, AR 72701, USA; nqharris@uark.edu (N.H.); cxm066@uark.edu (C.M.); 2 Center for Advanced Surface Engineering, University of Arkansas, Fayetteville, AR 72701, USA

**Keywords:** two-photon lithography, multi-material fabrication, 3D microvasculature, perfusion, neurovascular modeling, blood-brain barrier

## Abstract

Perfusable microvasculature is critical for advancing in vitro tissue models, particularly for neural applications where limited diffusion impairs organoid growth and fails to replicate neurovascular function. This study presents a versatile fabrication platform that integrates mesh-driven design, two-photon lithography (TPL), and modular interfacing to create multi-material, perfusable 3D microvasculatures. Various 2D and 3D capillary paths were test-printed using both polygonal and lattice support strategies. A double-layered capillary scaffold based on the Hilbert curve was used for comparative materials testing. Methods for printing rigid (OrmoComp), moderately stiff hydrogel (polyethylene glycol diacrylate, PEGDA 700), and soft elastomeric (photocurable polydimethylsiloxane, PDMS) materials were developed and evaluated. Cone support structures enabled high-fidelity printing of the softer materials. A compact heat-shrink tubing interface provided leak-free perfusion without bulky fittings. Physiologically relevant flow velocities and Dextran diffusion through the scaffold were successfully demonstrated. Cytocompatibility assays confirmed that all TPL-printed scaffold materials supported human neural stem cell viability. Among peripheral components, lids fabricated via fused deposition modeling designed to hold microfluidic needle adapters exhibited good biocompatibility, while those made using liquid crystal display-based photopolymerization showed significant cytotoxicity despite indirect exposure. Overall, this platform enables creation of multi-material microvascular systems facilitated by TPL technology for complex, 3D neurovascular modeling, blood–brain barrier studies, and integration into vascularized organ-on-chip applications.

## 1. Introduction

Three-dimensional (3D) in vitro models are increasingly essential for investigating human brain development, disease mechanisms, and drug responses. Compared to traditional two-dimensional (2D) cultures, which fail to replicate spatial organization and physiological interactions, 3D models better mimic the complex microenvironments of native tissues [1,2]. Advances in organoids, hydrogel scaffolds, and 3D bioprinting have expanded neural tissue modeling capabilities [3,4,5]. However, a critical limitation persists: the lack of perfusable microvasculatures.

The brain’s microvascular network plays an indispensable role in maintaining tissue viability and homeostasis [6,7]. In the absence of vascularization, brain organoids are restricted in size and often develop necrotic cores due to limited oxygen and nutrient diffusion [8,9,10]. Human brain capillaries are exceptionally narrow (7–10 µm in diameter) and densely spaced (~25 µm apart), forming tortuous pathways essential for efficient exchange and blood–brain barrier (BBB) support [11,12]. The BBB restricts passage of nearly all large molecules and up to 98% of small molecules, presenting additional challenges for central nervous system drug delivery [13]. Moreover, the lack of physiological flow and endothelial interaction in existing models limits their utility in replicating neurovascular dynamics and investigating disease pathogenesis [14,15,16].

To address these limitations, various strategies have been explored to introduce microvasculature into 3D models. Self-organized endothelial networks offer biological integration but often suffer from poor reproducibility and lack physiological organization [17,18,19,20]. Traditional microfluidic platforms provide precise flow control, yet their planar, oversized channels fail to emulate the complex and capillary-scale architecture of native brain microvasculature [20,21,22,23,24,25].

Two-photon lithography (TPL) offers sub-micron resolution and user-defined 3D geometries, making it a promising approach for constructing capillary-scale scaffolds [26,27]. Prior studies have used TPL to fabricate microvascular and BBB models [28,29]. However, these models primarily employ rigid photoresists and simple straight tube designs, resulting in quasi-2D structures with limited spatial complexity. They are unable to replicate the tortuous, branching, and arbitrarily oriented capillary geometries required to truly mimic brain microvasculature in three dimensions. Furthermore, they lack surrounding 3D lattice structures that enable neural cells to distribute and organize naturally in three dimensions around perfusable capillaries—a requirement for neurovascular unit (NVU) formation and functional neurovascular modeling. Practical issues with perfusion interfaces, often relying on bulky, non-integrated connectors, also hinder their adoption for long-term and biologically integrated studies.

In this study, we introduce a versatile and modular platform that addresses these challenges through three key innovations. First, a mesh-based design workflow in Rhinoceros 3D enables generation of arbitrarily routed 3D capillary scaffolds with tunable pore geometries and integrated mesoscale supports. These lattices not only stabilize soft capillary structures but also provide 3D environments to support neural cell attachment, distribution, and organization. Second, multi-material TPL fabrication using rigid (OrmoComp), intermediate stiffness (polyethylene glycol diacrylate, PEGDA 700) and soft elastomeric (IP-polydimethylsiloxane (PDMS)) materials enables tuning of scaffold mechanical properties across a wide range, balancing geometric fidelity and biological compliance. Rigid OrmoComp ensures precision and stability, PEGDA 700 offers a synthetic hydrogel to mimic a natural extracellular matrix environment, and IP-PDMS enables architected structures with unmatched softness. Third, a simple microfluidic interface based on heat-shrink tubing and hypodermic needles eliminates bulky fittings and facilitates leak-free perfusion. Together, these advances establish a customizable platform capable of supporting perfusable, biomimetic microvasculature systems for neural cell culture, neurovascular co-culture, and advanced brain-on-chip models. This system holds broad potential for applications in neurodegenerative disease modeling, BBB permeability studies, and vascularized organoid development.

## 2. Materials and Methods

### 2.1. Path-Adaptable Microcapillary Design Platform

**Figure 1:**
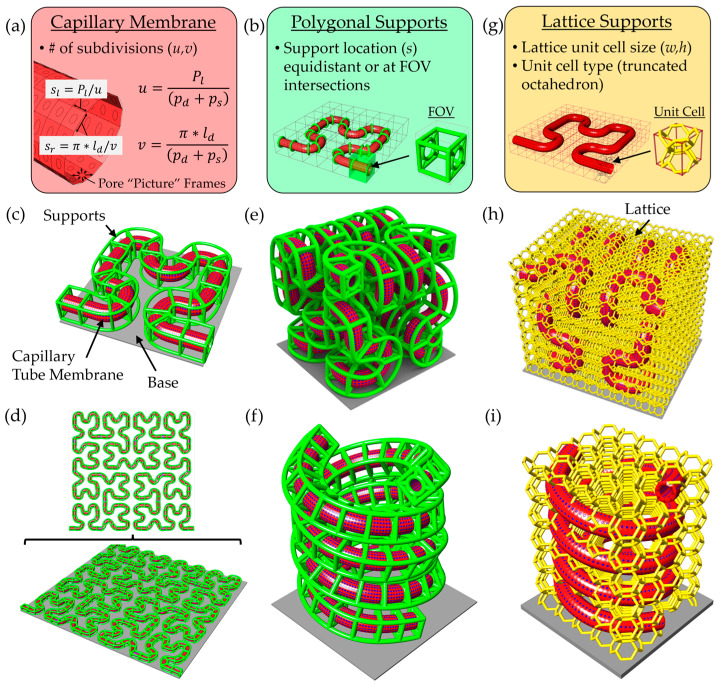
Design workflow and support strategies for versatile microcapillary scaffolds design platform. (**a**) Capillary membrane pore generation, including equations used for calculating the surface subdivisions to achieve the desired number of subdivisions and their side lengths. Capillary membrane geometry is shown in red. (**b**) Polygonal support structure design shown in green. Two-dimensional examples of the polygonal support strategy are shown, including (**c**) 2D Hilbert curve with supports at FOV intersections and (**d**) a fourth-degree planar Hilbert microcapillary scaffold. Three-dimensional designs using the polygonal support strategy include (**e**) 3D second-degree Hilbert curve and (**f**) double-helix designs with equidistant supports. Notably, the 3D polygonal support designs cannot be fabricated because they lack strong connection to the substrate at the base. (**g**) Schematic of lattice-based support method for 3D capillary designs based on a truncated octahedron unit cell shown in gold. Design examples using the lattice-based support method include (**h**) 3D second-degree Hilbert scaffold and (**i**) double-helix designs.

To generate complex and physiologically relevant microcapillary geometries, we developed a versatile scaffold design pipeline using modeling software made by Rhinoceros 3D called Rhino 7 along with a plugin called Grasshopper. This platform enables rapid generation of microcapillary models along arbitrary paths, with tunable parameters including lumen diameter, pore diameter, pore spacing, wall thickness, and support beam thickness. Microcapillary paths are imported from comma-separated value (.csv) files containing 3D coordinates, which define a Non-Uniform Rational B-Spline (NURBS) curve used as the central axis of the capillary geometry. Pores in the surface of the microcapillary’s cylindrical lumen are created using mesh-based operations rather than traditional model tree-based CAD modeling. A parametric domain subdivision of the cylindrical capillary surface sets the spacing and location of pores (Figure 1a).

Two equations control the pore distribution along the capillary tube surface, including:

(1) $$u=\frac{P_{l}}{\left(p_{d}+p_{s}\right)}$$

(2) $$v=\frac{{\pi *l}_{d}}{\left(p_{d}+p_{s}\right)}$$

where *u* in Equation (1) defines the number of subdivisions along the capillary tube path length (*P_l_*) based on the desired pore diameter (*p_d_*) and desired pore spacing (*p_s_*). Similarly, *v* in Equation (2) defines the number of subdivisions in the circumferential direction for the desired lumen diameter (*l_d_*), pore diameter, and pore spacing. Therefore, the side lengths of each surface subdivision are determined using the following:

(3) $$s_{l}=\frac{P_{l}}{u}$$

(4) $$s_{r}=\frac{{\pi *l}_{d}}{v}$$

where Equation (3) defines the length along the capillary membrane path direction (*s_l_*), and Equation (4) defines the length along the capillary membrane circumferential direction (*s_r_*). A frame enclosing each pore is created using *Weaverbird’s Picture Frame* function, with an offset thickness stipulated by the user. The offset thickness of the frame was set equal to half the pore separation distance to generate a pore with the desired diameter. After this step the mesh is thickened to desired capillary membrane thickness and smoothed using *Weaverbird* and *Catmull–Clark* functions.

To suspend the capillary membrane off the printing substrate, we implemented two scaffold support strategies: polygonal support and lattice-based support structures. The polygonal support method adds beams arranged in polygon shape around the capillary, connected via diagonal struts to a circular collar that wraps around the perimeter of the capillary. Users can define support spacing, polygon type, and rotational offset. Supports may be uniformly distributed or strategically placed at predicted field-of-view (FOV) transitions based on the TPL system’s lens and slice settings (Figure 1b). A Hilbert curve-based microcapillary illustrates this method, with green support beams placed at FOV intersections (Figure 1c). The design platform is scalable, easily accepting larger and more complicated 2D paths (Figure 1d). Examples of 3D configurations using this support strategy are shown, including Hilbert curves (Figure 1e) and double helices (Figure 1f).

However, this approach relies on the connection at the bottom to the base and cannot fully support arbitrary 3D curves. To address this, we employed a lattice-based strategy using a voxelized bounding box generated via the Crystallon plugin (Figure 1g), which ensures the connections among all unit cells at the box boundary. A truncated octahedron unit cell was selected for its high porosity and structural connectivity. Importantly, lattice beams intersecting the capillary volume were removed using the *PointInBrep* function, and the remaining struts were converted to solid pipe Brep objects with rounded caps, rendered in gold color. This support method not only enables fabrication of complex 3D geometries but also promotes 3D cellular integration. Examples using 3D Hilbert and double-helix capillaries demonstrate the method’s capabilities (Figure 1h,i). This flexible approach facilitates rapid tuning of architectural complexity, microcapillary spacing, and spatial coverage for a range of in vitro tissue modeling applications.

### 2.2. TPL Materials and Resin Preparation

OrmoComp (Micro Resist Technology GmbH, Berlin, Germany) and IP-PDMS (Nanoscribe GmbH, Eggenstein-Leopoldshafen, Germany) resins were used as received from the manufacturers. These materials provided rigid and elastomeric mechanical properties, respectively, and were used to fabricate scaffold regions requiring high geometric fidelity (OrmoComp) or flexible, tissue-mimicking characteristics (IP-PDMS).

PEGDA with a molecular weight of 700 kDa (PEGDA 700, Sigma-Aldrich, St. Louis, MO, USA) was selected as a moderately stiff material to balance geometric stability and biological relevance. PEGDA 700 was filtered through a MEHQ column filter (Sigma-Aldrich, St. Louis, MO, USA) to remove polymerization inhibitors, then mixed with 1% *w*/*v* Irgacure 819 photoinitiator (Sigma–Aldrich, St. Louis, MO, USA). The mixture was vortexed at 2500 rpm for 1 min to ensure homogeneity. Air bubbles were removed by vacuum degassing prior to TPL printing.

By combining rigid, intermediate, and elastomeric resins, this material system allowed tuning of scaffold mechanical properties and supported fabrication of perfusable microcapillary scaffolds for versatile cell culture applications.

### 2.3. TPL Fabrication of Microcapillary Designs

Microcapillary scaffolds were fabricated using TPL to achieve high-resolution, geometrically precise structures suitable for integration into microfluidic platforms. Scaffold models, including capillaries, support lattices, and base geometries, were designed in Rhinoceros 3D and exported as STL files. Test scaffolds were initially printed with a range of lumen diameters (40–80 µm), and a 40 µm-diameter capillary chip based on two stacked Hilbert curves was used for structure testing and flow validation. A flat base was added below the scaffold to help ensure adhesion in aqueous conditions.

All scaffold component models were converted to mesh format, and mesh density was reduced by 95% using the *ReduceMesh* command in Rhino 7 to minimize file size for efficient slicing in DeScribe software (version 2.5.3, Nanoscribe GmbH). The models were then sliced in DeScribe using optimized parameters: a slice thickness of 1.0 µm, hatch distance of 0.2 µm, and six contour lines. Solid infill was applied to ensure structural integrity, and stitching across adjacent fields of view (FOVs) was controlled using a 15° stitching angle and 5 µm overlap to minimize discontinuities.

**Figure 2:**
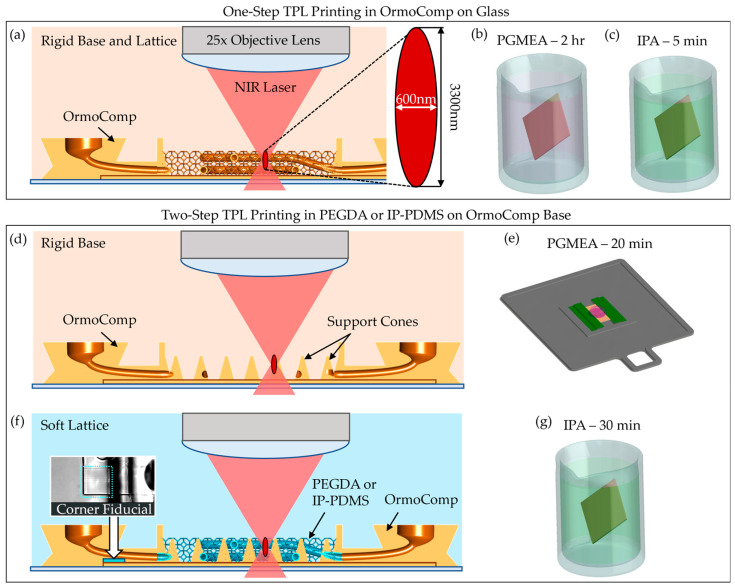
Three-dimensional printing of microcapillary scaffolds using TPL. (**a**) Single-step TPL printing process using OrmoComp (shown in orange) for both the base and the vasculature components. Uncrosslinked OrmoComp was removed using baths of (**b**) PGMEA (shown in purple) and (**c**) IPA (shown in green). (**d**) First stage of dual-step TPL printing process using OrmoComp for the base components. (**e**) In-place development of the OrmoComp using PGMEA. (**f**) Stage two of dual-step TPL printing process using PEGDA 700 or IP-PDMS (shown in blue) to print the vasculature components. The inset shown is a top-down view of the corner fiducial mark printed for alignment after resin application. The fiducial mark is outlined using a dotted blue line. (**g**) PEGDA 700 and IP-PDMS were developed in IPA after printing.

Fabrication was performed on a Nanoscribe Photonic Professional GT+ system (Nanoscribe GmbH, Eggenstein-Leopoldshafen, Germany) equipped with a 25×, NA 0.8 immersion objective, operating in Dip-in Laser Lithography (DiLL) mode with a 780 nm femtosecond laser. A schematic of the DiLL printing process is shown in Figure 2a. Coverslips (12 mm diameter, No. 2 thickness) were cleaned sequentially with acetone, isopropanol, and deionized water, followed by oxygen plasma treatment. To promote scaffold adhesion, Ormoprime (Micro Resist Technology GmbH, Berlin, Germany) was spin-coated onto the coverslips at 4000 rpm for 1 min and baked at 150 °C for 5 min. The prepared coverslips were taped on all sides onto indium tin oxide (ITO)-coated glass slides for mounting. A focal offset of approximately 200 µm (glass slide thickness) was applied to target the focal plane ~8 µm inside the coverslip, ensuring uniform adhesion and printing consistency across the sample.

In TPL, a near-infrared (NIR) femtosecond laser is tightly focused to induce two-photon absorption in the resist. At the focal point, the simultaneous absorption of two photons initiates local crosslinking, forming a hardened voxel with a diameter of approximately 600 nm in the short axis and 3300 nm in the long axis [30]. Printing parameters were adjusted based on the material properties. For OrmoComp scaffolds, printing was conducted using a laser power of 40 mW and a scan speed of 80 mm/s. After printing, the structures were developed in a propylene glycol monomethyl ether acetate (PGMEA, VWR, Radnor, PA, USA) bath for 2 h, followed by a 5 min rinse in isopropanol (IPA, VWR, Radnor, PA, USA) (Figure 2b,c).

For soft materials, including PEGDA 700 and IP-PDMS, a dual-material develop-in-place workflow was implemented to prevent collapse during printing and post-processing. Initially, OrmoComp base structures with integrated nozzle ports were printed directly onto the coverslip and developed in PGMEA for 20 min, rinsed with IPA, and dried with nitrogen (Figure 2d,e). A large droplet of OrmoComp was required to avoid bubble forming in the second layer of the 3D-printed structure.

Next, PEGDA 700 or IP-PDMS resin was manually dispensed onto the developed OrmoComp base. Air bubbles were carefully removed with forceps to avoid potential printing defects. The sample was reloaded into the Nanoscribe system, and the objective was manually realigned using recorded focal height offsets. The alignment was further fine-tuned by printing fiducial marks at the corner of the scaffold to ensure accurate overlay (inset of Figure 2f). Capillary networks and lattice supports were then printed directly on the OrmoComp base, which contains cone structures strategically printed on the base to provide distributed support of the soft scaffolds. PEGDA 700 was printed using a laser power of 50 mW and a scan speed of 74 mm/s, while IP-PDMS was printed using a laser power of 40 mW and a scan speed of 50 mm/s.

Upon completion, scaffolds printed in PEGDA and IP-PDMS were developed in IPA for 30 min to remove uncrosslinked resin and air dried (Figure 2g). This dual-material fabrication process successfully integrated rigid and soft polymers into single scaffolds, enabling complex geometries with enhanced mechanical robustness while supporting soft capillary networks that resisted collapse during solvent development.

### 2.4. Microfluidic Interface

To establish perfusion through the TPL-printed microcapillary scaffolds, a modular microfluidic interface was engineered. This system utilized polyolefin heat-shrink tubing (McMaster-Carr, Douglasville, GA, USA) and 22-gauge hypodermic needles (Careach Direct, Seattle, WA, USA) to provide a compact, scalable, and cost-effective alternative to bulky commercial connectors that are often incompatible with delicate microscale architectures.

**Figure 3:**
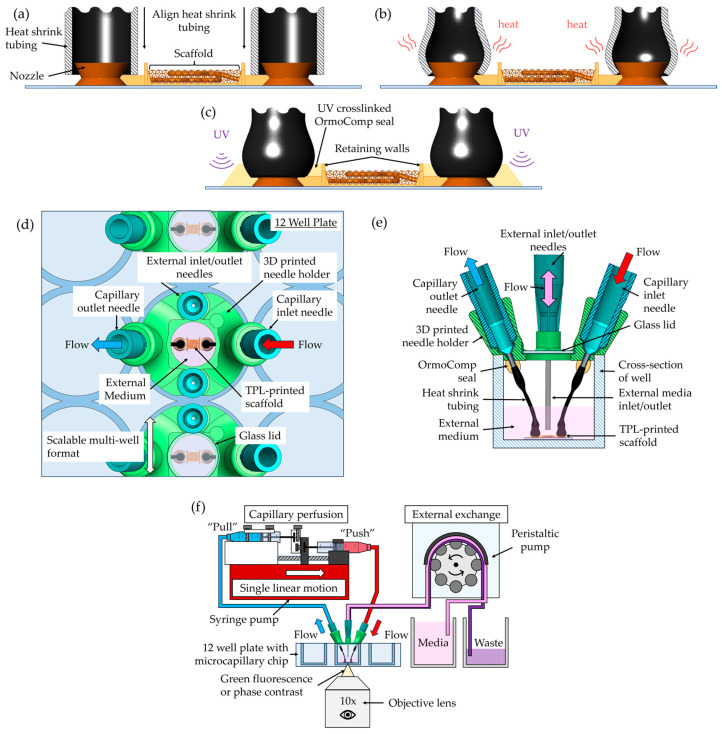
Microfluidic interface and pumping setup facilitated via heat-shrink tubing interface. Schematics of (**a**) heat-shrink tubing aligned to the TPL-printed nozzles without heat, (**b**) application of heat to shrink the tubing around the nozzles, and (**c**) UV crosslinking OrmoComp to seal the connection. (**d**) Top–down and (**e**) side views of design of the 3D-printed hypodermic needle holder. The 3D-printed needle holder is UV crosslinked with OrmoComp onto the top of the well of a 12-well plate. The TPL-printed scaffold is secured to the bottom of the well following forming the heat-shrink tubing connections. The hypodermic needles are then inserted into the heat-shrink tubing ends through the capillary needle inlet and secured in place via heat and OrmoComp resin. This creates a sealed interface for perfusion of the capillary membrane. A second set of needles is used to exchange the medium on the outside of the membrane. A removable glass lid is used to keep the chamber from evaporating. (**f**) Schematic of the pump setup is shown, including capillary perfusion via syringe pump and external exchange via peristaltic pump. Capillary perfusion is accomplished by modifying a linear syringe pump to perform push and pull actions in the same motion. The 12-well plate is observed in situ using a small-format microscope.

The assembly process is shown in Figure 3a–c. Vertical nozzle ports were designed directly into the OrmoComp scaffold base and were optimized to mate with 0.020-inch inner diameter heat-shrink tubing (Figure 3a). Following scaffold development, the tubing was manually fitted onto the nozzle ports and locally heated using a soldering iron for 1–2 s. This heating step caused the tubing to contract, creating a tight, press-fit seal against the nozzle surfaces (Figure 3b). To further secure the connection and prevent leakage, OrmoComp resin was applied around the tubing at the junction. A retaining wall was used to prevent resin from spreading into the microvasculature. The resin was cured under ultraviolet (UV) light at 100 mW/cm^2^ for 1 min (Figure 3c).

The entire assembly was then secured to the bottom of a standard 12-well plate using a small drop of OrmoComp resin, which was UV-cured to anchor the chip firmly in place. A custom 3D-printed holder was affixed to the top of the well using UV-cured OrmoComp. The top–down view is shown in Figure 3d, and the side view of single well is shown in Figure 3e. This holder included ports for two blunt 26-gauge hypodermic needles (Careach, 0.5-inch length), enabling external media exchange during experiments.

Flow system integration was achieved by inserting blunt 22-gauge hypodermic needles into the exposed ends of the heat-shrink tubing. After insertion, the heat-shrink tubing was locally heated with a soldering iron to shrink tightly around the needles, ensuring robust mechanical engagement. OrmoComp resin was then applied around the needle-tubing junction and UV-cured (100 mW/cm^2^, 1 min) to complete a leak-free seal.

The chip was covered with a removable 12 mm-diameter glass lid to fully enclose the chamber and maintain sterility. For perfusion experiments, the assembled device was connected via flexible tubing to a syringe pump operated in a push–pull configuration, allowing controlled, bidirectional flow through the scaffold (Figure 3f). This setup minimized pressure fluctuations and ensured gentle, continuous perfusion compatible with neurovascular models. An in situ imaging system (Etaluma LS560 microscope (Etaluma, San Diego, CA, USA) equipped with a custom 3-axis stage) was used to monitor perfusion in real time without disrupting incubator conditions (Figure 3e).

### 2.5. Sterilization and Scaffold Presoak

To ensure biocompatibility and minimize cytotoxic effects from fabrication residues, all microfluidic chip components underwent rigorous sterilization and presoak protocol prior to biological testing. This two-stage process was designed to remove leachable contaminants and condition scaffold surfaces for optimal cell interaction.

All chip materials, including TPL-printed scaffolds (OrmoComp, PEGDA 700, and IP-PDMS), heat-shrink tubing, 3D-printed parts (Siraya Tech Blu, Anycubic Transparent Green, and PLA), and tissue culture plastics, were sterilized for viability testing. All materials were first rinsed with 70% ethanol for 2 m. After air-drying in a laminar flow hood for 30 min, the materials were exposed to germicidal ultraviolet (UV) light for 1.5 h.

Following sterilization, a prolonged presoak process was implemented to further reduce potential cytotoxic leachates, such as residual photoinitiators and unreacted monomers. During the initial 2 days (Days 0–1), chips were rinsed at 1-h intervals for 3 h with phosphate-buffered saline (PBS) to remove soluble impurities. From Days 2 to 6, chips were washed daily and incubated in complete neural growth medium to allow surface equilibration and passivation. This presoak stage was critical for minimizing acute cytotoxic responses upon subsequent cell seeding.

The neural growth medium used during presoak was formulated to support neural stem cell health. It consisted of basal medium (Vesta Biotherapeutics, formerly Phoenixsongs Biologicals, Branford, CT, USA) supplemented with Neural StemCell Growth Base Supplement, laminin, basic fibroblast growth factor (bFGF), epidermal growth factor (EGF), and a proprietary neural culture factor. Gentamicin (37.5 mg/L, St. Louis, MO, USA) was included to prevent microbial contamination during extended incubation. All media components were sterile filtered using a 0.22 µm membrane and stored at 4 °C prior to use.

### 2.6. Surface Coating

To enhance cellular adhesion and prepare scaffold surfaces for neural cell culture, a sequential coating protocol using poly-D-lysine and laminin was applied. This approach improved scaffold biocompatibility and mimicked essential extracellular matrix features.

Scaffolds and controls (tissue culture plastic) were first incubated in a 5 µg/mL solution of poly-D-lysine (Thermo Fisher Scientific, Waltham, MA, USA) for 10 min at room temperature. This cationic polymer increases surface charge and promotes initial cell adhesion on synthetic and hydrogel-based materials. Following incubation, samples were rinsed three times with sterile, cell culture-grade water to remove unbound poly-D-lysine and ensure uniform surface coating.

Next, laminin (Thermo Fisher Scientific, Waltham, MA, USA) was applied at a concentration of 10 µg/mL and incubated for 2 h at room temperature. As a key extracellular matrix protein, laminin supports neuronal attachment, differentiation, and survival, making it critical for creating a cell-compatible microenvironment. After incubation, excess laminin was aspirated and samples were gently rinsed with sterile water to remove residual solution. Culture medium was then added to the wells to maintain hydration and prepare samples for cell seeding. To stabilize the coating and equilibrate the scaffolds to culture conditions, coated samples were placed in a humidified incubator for 1 h prior to cell plating.

### 2.7. Cytotoxicity Assay

The cytocompatibility of TPL materials and associated microfluidic chip materials was evaluated using a Live/Dead^®^ viability assay (Thermo Fischer Scientific, Waltham, MA, USA) with human hippocampal-derived neural stem cells (hNSCs; HIP-009, Vesta Biotherapeutics, Branford, CT, USA). These cells, chosen for their sensitivity to material-induced toxicity and relevance to neurobiological studies, provided an effective model for assessing material suitability for brain-on-chip applications.

Cells were seeded directly onto TPL materials (OrmoComp, PEGDA 700, and IP-PDMS) molded flat with a PDMS stamp and cured under 100 mW/cm^2^ UV for 5 min, chip materials (heat-shrink tubing and wells with hypodermic needle holder lids) and control substrates immediately after thawing from cryopreservation at a density of 4.5 × 10^5^ cells per well. Non-adherent cells and debris were removed by media exchange on Day 1 to ensure uniform attachment. Cultures were then maintained without further media changes until Day 3 under static conditions.

At the conclusion of the culture period, viability was assessed using the Live/Dead assay, which differentiates live and dead cells by fluorescence. Calcein AM (2 µM, Ex: 484 nm, Em: 517 nm) labeled viable cells green, while ethidium homodimer-1 (4 µM, Ex: 528 nm, Em: 617 nm) stained non-viable nuclei red. Samples were incubated in staining solution for 20 min at 37 °C before imaging.

Fluorescence imaging for cytotoxicity assay and scaffold material fluorescence analysis was performed on an Olympus IX-70 inverted microscope (Olympus, Center Valley, PA, USA) equipped with filter cubes: U-MNUA2 for blue (Ex: 365 nm, Em: 440 nm), U-MNIBA3 for green (Ex: 480 nm, Em: 530 nm), and U-MRFPHQ for red (Ex: 545 nm, Em: 597 nm) (Hunt Optics, Pittsburgh, PA, USA). Images were captured using a CoolSnap K4 camera (Photometrics, Huntington Beach, CA, USA) controlled by MicroManager software (version 1.4.23). Exposure times were standardized to 1000 ms across all channels to ensure consistency.

Quantitative analysis was conducted by imaging three independent regions per sample at 4× magnification, yielding nine images per material group. Images were processed using a custom MATLAB (2024a) script for automated segmentation and initial cell counting. The script utilized imbinarize() and label2rgb() functions to identify live and dead cells from fluorescence images. This was followed by manual correction with a secondary script to verify accuracy. The correction script utilized the ginput() function to provide the user with a graphical user interface (GUI) to correct live cells marked dead, dead cells marked live, and live or dead cells that were missed in the automatic count. In total, viability calculations were based on approximately 116,000 cells.

### 2.8. PEGDA Mechanical Property Characterization

While mechanical data for OrmoComp and IP-PDMS produced by TPL are available in the literature, equivalent characterization for PEGDA 700 formulated with 1% Irgacure 819 photoinitiator had not been reported. As this formulation was specifically utilized for nanoprinting in this study, direct measurement of its mechanical properties was necessary.

Test specimens were printed using TPL as square prisms (200 µm × 200 µm × 10 µm) onto methacrylate-treated glass coverslips (3 h in 3-(Trimethoxysilyl)propyl methacrylate, Sigma-Aldrich, St. Louis, MO, USA) to ensure adhesion during testing. Nanoindentation was conducted under ambient conditions using a Hysitron TriboIndenter (Bruker, Eden Prairie, MN, USA) equipped with a spheroconical diamond probe (5 µm tip radius). A linear load–unload rate of 10 µN/s was applied up to a peak load of 250 µN, with a 5 s hold at maximum load.

The Young’s modulus and hardness of PEGDA 700 were calculated based on the nanoindentation load-displacement curves using the Oliver–Pharr method [31]. Reported values reflect the mean ± standard deviation from three indentations.

### 2.9. Geometry Registration Analysis

A geometry registration analysis was performed to quantitatively assess scaffold fidelity by comparing fabricated structures to their original digital designs. Deviations were expected to arise from two sources: inaccuracies in the printing process and structural deformation during post-fabrication steps (such as solvent development and drying). As imaging was performed only after development, the measured deviations reflect the combined contributions of both factors.

Binary reference masks representing the intended geometries were generated from the original design files. Top–down renders of scaffold models were exported from Rhinoceros 3D and processed in ImageJ (version 1.54g) to produce binarized black-and-white target images. These served as the reference standard for assessing print fidelity.

Experimental masks were created from scanning electron microscopy (SEM, Vega3, Tescan, Warrendale, PA, USA) images of the printed scaffolds. SEM images were first aligned and cropped using the flat features on the edge of the scaffolds as a repeatable reference. SEM images were then processed to enhance feature visibility, including a global contrast enhancement of 30%. For regions with faint features, particularly at the image edges, localized adjustments were manually applied to improve clarity. The processed images were then binarized to isolate capillary membrane and lattice structures.

To address areas where thresholding alone did not sufficiently capture scaffold features, edge detection using the “Find Edges” function in ImageJ was combined with manual editing to fill in missing regions. Cone support structures were digitally subtracted to ensure that only relevant scaffold features remained for analysis.

Target and experimental masks were then merged using color channels to generate composite registration maps. The target design was assigned to the green channel and the experimental print to the red channel. Therefore, in the resulting maps, yellow indicated overlapping features, green indicated missing scaffold material, red indicated deformed printed material, and black indicated void regions. The overlapping areas were analyzed. This registration method allowed systematic evaluation of printing fidelity, capturing combined deviations resulting from both printing imprecision and post-processing deformation.

### 2.10. Diffusion and Particle Flow Testing

Functional testing of the assembled chips was conducted using fluorescein-labeled Dextran (Thermo Fischer Scientific, Waltham, MA, USA) and 1 µm fluorescent polystyrene beads (Sigma-Aldrich, St. Louis, MO, USA). For diffusion experiments, Dextran in phosphate-buffered saline (PBS) was perfused through the capillary scaffolds using a syringe pump (Pump Systems Inc., Franklin, NH, USA) configured with an auxiliary syringe holder to enable push–pull flow (Figure 3f). A peristaltic pump (Ismatec, Hood River, OR, USA) was used to circulate medium in the external chamber surrounding the scaffold. The syringe pump was operated at a flow rate of 1 µL/min, and diffusion across the capillary membrane was imaged in situ using an Etaluma LS560 microscope. The camera exposure time was set to 5.1 ms to match calibration conditions for Dextran fluorescence.

To assess flow speed, particle image velocimetry (PIV) was performed by perfusing 1 µm green fluorescent beads through a 1 mm-long solid-walled channel with a 40 µm diameter—equivalent to the lumen geometry of the scaffold. This open-channel configuration was used to avoid clogging observed in earlier scaffold-based attempts. Beads were suspended in water and driven through the channel at 10 nL/min using the same syringe pump in unidirectional push mode. Timelapse videos were acquired and analyzed using a custom MATLAB script utilizing the ginput() GUI to extract bead trajectories between individual video frames and calculate flow velocity by dividing by the camera exposure rate.

## 3. Results

### 3.1. Scaffold Fabrication with Polygonal and Lattice Support Strategies

**Figure 4:**
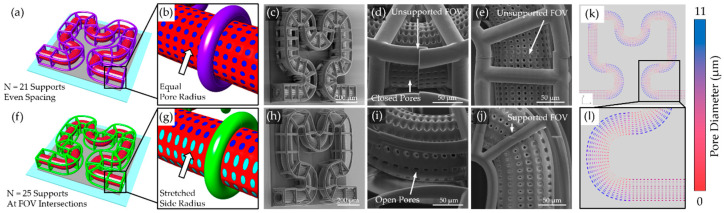
Design optimization for two-dimensional microvasculatures printed in OrmoComp. (**a**) Three-dimensional model of a second-degree Hilbert curve vasculature design with 21 support rings evenly spaced along the path length of the curve (shown in purple). (**b**) Close-up view of the pores (shown in blue). (**c**) Isometric SEM image showing the test print. SEM micrographs showing (**d**) misaligned sections due to unsupported geometry at the FOV intersections and closed pores on the sides, and (**e**) top view of misaligned sections at FOV intersections. (**f**) Three-dimensional model of the same Hilbert curve design using 25 support rings placed at FOV intersections (shown in green). (**g**) Close-up view of elongated pores on the sides of the vasculature model (elongated pores shown in cyan). (**h**) SEM micrographs showing the isometric view of the test print, (**i**) open pores along the sides of the vasculature membrane, and (**j**) the top view of aligned geometry at FOV intersections due to appropriately placed supports. Renders (**k**,**l**) of pore diameter distribution along Hilbert curve and straight curve paths are shown.

Support strategies for microcapillary fabrication were evaluated using a planar Hilbert curve scaffold design. This model, shown in Figure 4a, featured a lumen diameter of 80 µm, pore diameter of 5 µm (Figure 4b), pore spacing of 5 µm, membrane thickness of 15 µm, and polygonal support rings with a 20 µm diameter positioned along the capillary path. Initial fabrication trials using this configuration (Figure 4c–e) revealed critical limitations. Although the overall scaffold geometry was achieved, closed pores were observed along the lateral sides of the capillary membrane, and significant misalignment occurred at FOV intersections. These issues were most pronounced in unsupported regions where capillary sections were suspended without reinforcement.

To address these problems, several design modifications were implemented. The pore was elongated in the circumferential direction by approximately 1.5× to keep the pores from closing. In addition, the support beams were repositioned to align with predicted FOV intersections (Figure 4f,g). By making these changes, the structure was able to be redesigned with reduced beam diameters of 15 µm to reduce material usage and increased pore spacing to 8 µm separation for added stability. These adjustments improved scaffold fidelity. SEM imaging confirmed preservation of pore openings and reduced stitching misalignment (Figure 4h–j). However, uniformity of pore sizes remains a challenge. Analysis of designed pore diameters along the scaffold path (Figure 4k,l) showed significant variability, with pores constricting on interior curves and widening on exterior curves. Additionally, the polygonal support structures rely on direct connection to the base, limiting the vasculature format to 2D curves parallel to the surface.

**Figure 5:**
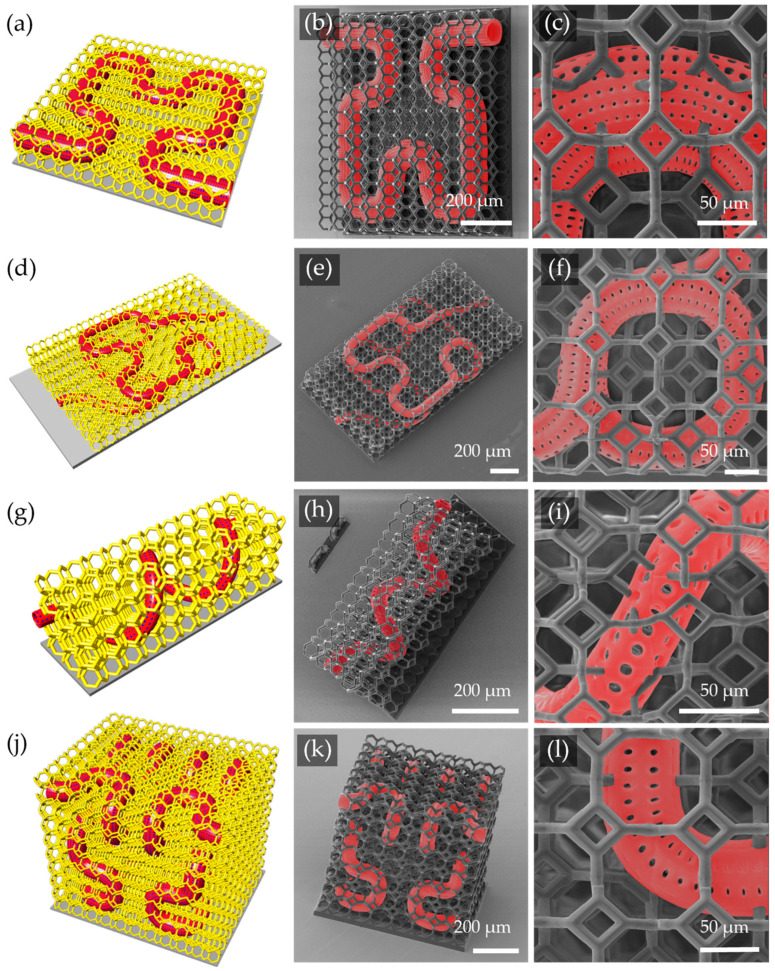
Design and TPL test prints of 2D and 3D microvascular scaffolds with integrated lattice support, all fabricated using OrmoComp. SEM micrographs are colorized to highlight vasculature geometry in red. (**a**–**c**) Two-dimensional Hilbert curve: (**a**) Rhino model, (**b**) isometric SEM view, and (**c**) close-up of pores. (**d**–**f**) Stacked 2D Hilbert curves: (**d**) Rhino model, (**e**) isometric SEM view, and (**f**) pore details. (**g**–**i**) Helical scaffold: (**g**) Rhino model, (**h**) isometric SEM view, and (**i**) pore close-up. (**j**–**l**) Three-dimensional second-degree Hilbert curve: (**j**) Rhino model, (**k**) isometric SEM view, and (**l**) pore close-up.

To overcome these constraints, a lattice support strategy was introduced to provide continuous reinforcement throughout the scaffold volume. A lattice-supported version of the planar Hilbert curve scaffold was fabricated using the same overall geometric parameters (Figure 5a–c). It was shown that, due to higher support density, the support beam diameter could be reduced to 10 µm without affecting print fidelity. Colorized SEM micrographs, used to improve visualization of scaffold geometry, confirmed robust 3D support and substantially improved structural stability, particularly at FOV intersections where deformation had previously been problematic.

The versatility of lattice supports was further demonstrated through fabrication of additional capillary scaffold designs. These included a vertically stacked two-layer Hilbert curve with a lumen diameter of 50 µm (Figure 5d–f), a 3D helical scaffold with a 40 µm lumen diameter (Figure 5g–i), and a second-order 3D Hilbert curve with a 50 µm lumen diameter (Figure 5j–l). All scaffolds were printed using OrmoComp and exhibited no major defects, confirming that the lattice support strategy enabled reliable fabrication of complex three-dimensional microvascular architectures.

### 3.2. Multi-Material Scaffold Fabrication, Characterization, and Geometric Fidelity

Following validation of scaffold designs using OrmoComp, multi-material printing trials were conducted to investigate the feasibility of fabricating microcapillary scaffolds from materials with differing mechanical properties. OrmoComp, PEGDA 700, and IP-PDMS were selected to represent rigid, hydrogel, and elastomeric classes, respectively. These materials span a wide Young’s modulus spectrum and address different demands for rigidity, elasticity, and biomimicry. While OrmoComp and IP-PDMS had previously reported mechanical properties under relevant processing conditions (~1.27 GPa [32] and 350 kPa–17.5 MPa Young’s modulus [33], respectively), PEGDA 700 formulated with 1% Irgacure 819 photoinitiator had not been characterized. Thus, direct mechanical testing was required.

PEGDA 700 square prisms (200 × 200 × 10 µm) were printed onto methacrylate-treated glass coverslips and tested using nanoindentation. The measured Young’s modulus in dry condition was 30.7 ± 1.2 MPa, with a hardness of 8.3 ± 0.4 MPa. These values positioned PEGDA 700 between OrmoComp and IP-PDMS in elasticity, confirming its moderate rigidity and potential for scaffold fabrication. However, as expected, PEGDA 700 and IP-PDMS posed more significant challenges during fabrication due to their lower moduli and higher propensity for deformation during solvent development and drying.

**Figure 6:**
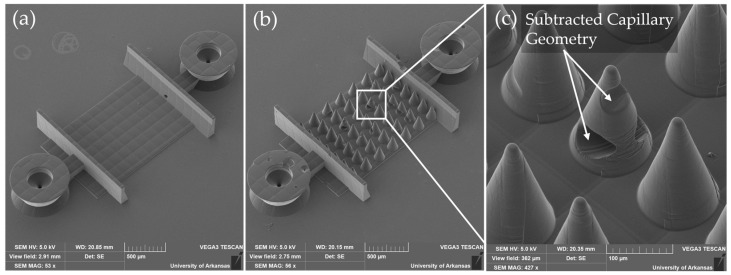
SEM micrographs of test-printed OrmoComp bases. (**a**) Flat base without mesoscale supports, (**b**) base with cone supports, and (**c**) close-up view of the cone supports. The capillary tube membrane geometry was subtracted from the cones to ensure the chips could still be perfused.

**Figure 7:**
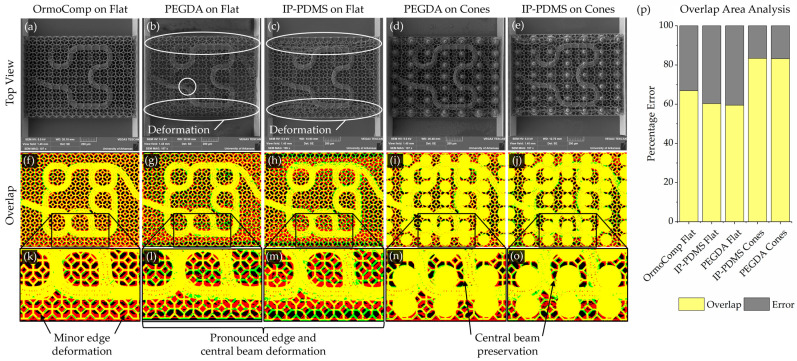
SEM imaging and registration analysis of microcapillary scaffolds fabricated from different materials with and without cone supports. (**a**–**e**) SEM images of scaffolds made from OrmoComp, PEGDA 700, and IP-PDMS on flat or cone-supported bases. (**f**–**j**) Overlap images comparing SEM results to Rhino-generated binary masks. Yellow = correct match; green = missing geometry; red = excess geometry; black = correct void. (**k**–**o**) Close-up views showing edge deformation on flat bases and improved geometry control with cone supports. (**p**) Quantitative registration analysis showing overlap (yellow) versus total print error (combined red and green).

To enable rigorous comparative analysis, scaffolds using the layered Hilbert–curve chip design shown in Figure 5d with a 40 µm lumen diameter, 5 µm pore diameter, 8 µm pore spacing, 10 µm membrane thickness, and 10 µm lattice beam diameters were fabricated across all materials. OrmoComp scaffolds were first printed directly on flat bases (Figure 6a) and demonstrated good printing fidelity with minor edge deformation (Figure 7a). In contrast, PEGDA 700 and IP-PDMS scaffolds printed on flat bases (Figure 7d,g) exhibited pronounced collapse, especially at curved regions and scaffold edges.

To mitigate these issues, mesoscale cone support structures were employed. The cone’s surface, shown in Figure 6b, enables geometry to be supported in the vertical direction. These OrmoComp-printed cones featured minimum taper angles of 15°, which were determined to be necessary to prevent printing errors caused by vertical and overhanging geometry blocking the laser path. The capillary path was subtracted from the cone’s geometry (Figure 6c) to ensure the cones did not block perfusion.

The cones provided critical support to stabilize soft materials during both printing and development. By printing onto cone-supported OrmoComp bases, both PEGDA 700 and IP-PDMS scaffolds displayed substantially improved outcomes (Figure 7d,e). The conical supports minimized deformation in the central area, though defects were still observed near the edges.

Geometric registration maps were generated to visualize scaffold deviations. Color-coded overlays, shown in Figure 7, revealed key deformation patterns. Yellow indicated correct overlap, green denoted missing material, red highlighted deformed geometry, and black represented void regions. Notably, excess material was predominantly located at scaffold edges, where deformation during drying was most pronounced. OrmoComp showed the highest fabrication accuracy on the flat base. The cone supports helped improve the outcomes of the IP-PDMS- and PEGDA-printing strategies, resulting in even better fidelity than using OrmoComp alone, underscoring the importance of appropriate support methods for softer material architectures.

### 3.3. Microfluidic Chip Sealing and Integration for Flow Testing

**Figure 8:**
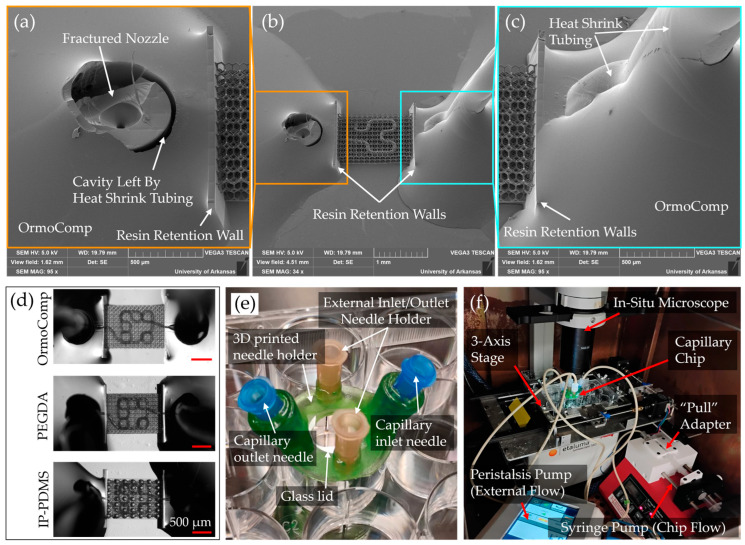
Fabrication and testing setup for capillary chips. (**a**) Close-up view of a nozzle with the tubing fractured away. (**b**) Overall view of an OrmoComp chip. (**c**) Close-up view of the nozzle with the tubing sealed in place with OrmoComp. (**d**) Assembly of heat-shrink tubing onto OrmoComp, PEGDA 700, and IP-PDMS scaffolds. (**e**) Overview of fabricated chip and hypodermic needle holder. (**f**) In situ testing setup, including a small-format microscope equipped with a custom three-axis stage, a syringe pump equipped for both push and pull functions, and a peristaltic pump for external flow for media exchange.

The integrity of the microfluidic sealing strategy was evaluated through SEM, and integration into a flow test setup is shown (Figure 8). During disassembly tests, removal of the heat-shrink tubing fractured the printed nozzle structure itself (Figure 8a,b). This fracture was most likely caused by compressive forces exerted between the tubing and the nozzle when removing the heat-shrink tube, indicating that a mechanically durable and leak-resistant bond had been formed. Importantly, examination of nozzle cross-sections (Figure 8b,c) confirmed that the OrmoComp resin seal was well confined within retention walls and did not extend into the scaffold lattice region, preserving open perfusion pathways essential for subsequent flow experiments.

Assembly of the completed microfluidic chip is shown in Figure 8d,e. The design utilized hypodermic needles as the inlet and outlet that were inserted into heat-shrink tubing. The needles were UV-cured securely inside the 3D printed needle holder using OrmoComp, enabling capillary perfusion ports and external ports for media exchange outside the capillary. The chip was housed within a standard 12-well plate with a removable glass lid (Figure 8e). This configuration maintained the chip sterility while enabling optical imaging access.

To integrate the chip into flow testing, it was connected to a syringe pump configured in push–pull mode for continuous flow and a peristaltic pump for external media exchange within the well volume. Mounted on an Etaluma LS560 microscope equipped with a custom three-axis stage, the assembled chip allowed in situ visualization of perfusion pathways during operation without disrupting incubator conditions (Figure 8f).

### 3.4. Evaluation of Scaffold Dimensional Stability, Diffusion Transport, and Perfusion Performance

**Figure 9:**
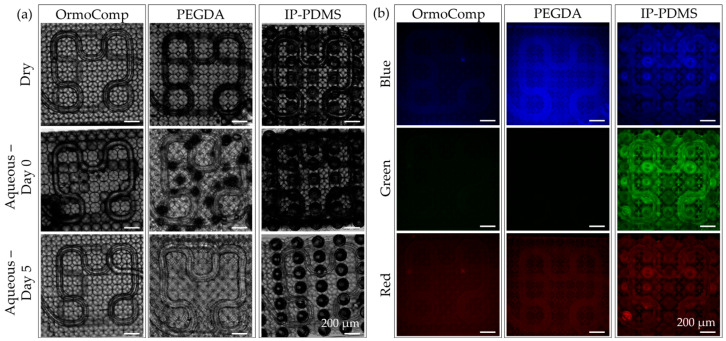
Assessment or OrmoComp, PEGDA 700, and IP-PDMS scaffolds under cell culture imaging conditions. (**a**) Brightfield images of dry and aqueous conditions. (**b**) Dry fluorescence imaging tests using blue, green, and red filter cube sets.

Following chip fabrication and integration into the perfusion setup, the system was evaluated for integrity under cell culture-like conditions. The first assessment focused on scaffold integrity after immersion in aqueous media. Brightfield imaging of dry and hydrated scaffolds revealed distinct responses across materials (Figure 9a). OrmoComp and IP-PDMS scaffolds demonstrated excellent dimensional stability, maintaining their original geometries without visible deformation after immersion. In contrast, PEGDA 700 scaffolds exhibited visible distortion of both capillary membranes and lattice supports. Notably, this deformation proved largely reversible. After five days of continuous incubation, PEGDA 700 scaffolds gradually returned to near-original geometry, suggesting that the swelling was driven primarily by heterogeneous medium uptake across the lattice. Additionally, air bubbles initially trapped within the lumen of all scaffold types were passively released during this equilibration period. IP-PDMS retains the greatest amount of air initially, while PEGDA and OrmoComp trap relatively less air volume.

Next, scaffold autofluorescence was evaluated, as optical clarity and low background signals are essential for fluorescence-based biological imaging. Dry scaffolds were imaged using standard blue, green, and red filter cubes (Figure 9b). OrmoComp exhibited the lowest autofluorescence across all channels, confirming its suitability for high-resolution fluorescence imaging. PEGDA 700 scaffolds displayed intense autofluorescence in the blue channel, while IP-PDMS showed moderate autofluorescence predominantly in the green channel, with noticeable autofluorescence also seen in blue and red channels. These observations underscore the importance of careful fluorophore selection when performing immunochemical staining or live imaging, particularly for scaffolds fabricated from PEGDA 700 and IP-PDMS.

**Figure 10:**
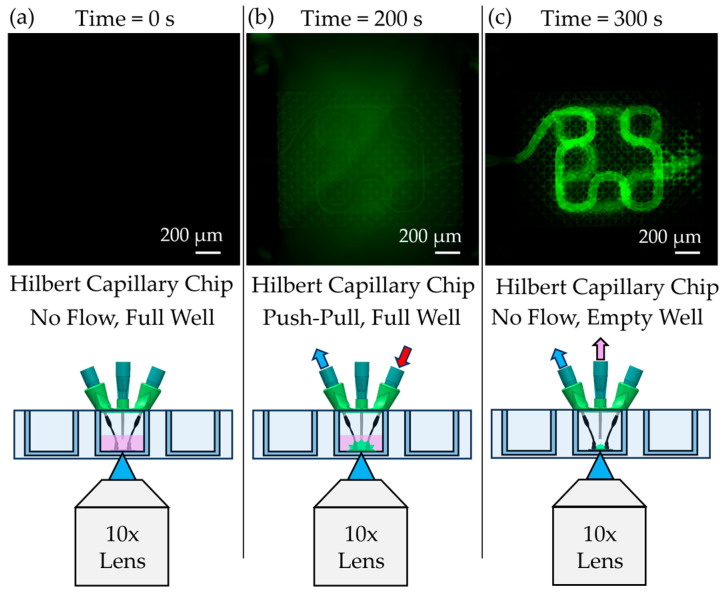
Dextran diffusion experiments in OrmoComp chips. (**a**) Fluorescence image prior to Dextran diffusion. (**b**) Diffusion of Dextran using the push–pull method. (**c**) Extraction of Dextran through membrane in chip, showing interior of double-layered Hilbert curve chip.

Molecular transport was then assessed using fluorescein-labeled Dextran to validate diffusion across the capillary membrane (Figure 10). The schematics of each testing condition are shown in the bottom row of the figure. An initial baseline image prior to flow confirmed the absence of background fluorescence (Figure 10a). After 200 s of perfusion, Dextran diffused from within the capillary lumen into the surrounding medium, as indicated by increased fluorescence intensity outside the membrane (Figure 10b). Subsequently, the Dextran was withdrawn through the outlet port using the push–pull flow configuration and the outlet for the external media. During this phase, a reverse concentration gradient caused Dextran to be reabsorbed into the capillary lumen (Figure 10c, green region), demonstrating the scaffold’s ability to support bidirectional diffusion across the membrane.

**Figure 11:**
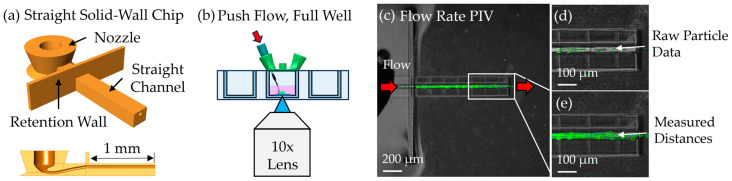
Particle flow speed measurement. (**a**) Three-dimensional rendering of a solid-wall straight microchannel with a 40 µm inner diameter and 1 mm length, shown in isometric and cutaway views. (**b**) Schematic of the push–flow setup used to drive bead perfusion through the microchannel. (**c**) Maximum intensity projection of PIV time-lapse frames overlaid on phase contrast background, illustrating complete particle paths. (**d**) Example image of fluorescent particles in flow during a single frame. (**e**) Tracked particle paths plotted from the PIV analysis.

Finally, the ability of the printed scaffolds to support perfusion at biologically relevant flow speeds was evaluated using fluorescent polystyrene beads (1 µm diameter). Initial flow measurements in the microcapillary scaffold were limited by particle clogging. To address this, flow testing was performed using a straight microchannel (1 mm long, 40 µm in diameter) that replicated the lumen geometry of the scaffold (Figure 11a). To minimize clogging risk, a simplified open-channel configuration was used with one inlet and an open outlet leading into the well reservoir, as shown in the chip design schematic (Figure 11a). Flow was driven using a syringe pump operating in a unidirectional push configuration (Figure 11b).

Flow speed was measured using a custom MATLAB script for PIV. Video frames showing bead movement were extracted and compiled into a maximum intensity projection, i.e., a single image that captures the full trajectories of moving particles (Figure 11c). Individual bead positions across frames were manually tracked using a graphical interface, and their displacements were used to calculate velocity. A raw frame showing tracked bead positions is shown in Figure 11d, and the compiled trajectories from the full sequence are shown in Figure 11e.

At a flow rate of 10 nL/min, the average velocity of 27 tracked particles was 1.83 ± 0.56 mm/s. This falls within the physiological range of cerebral microvascular blood flow (0.1–9.4 mm/s) [34], confirming that the fabricated microchannel and interfacing system are capable of supporting biologically relevant flow conditions.

### 3.5. hNSC Viability and Cytotoxicity Testing

To evaluate the biocompatibility of all materials integrated into the microvascular scaffold and microfluidic chip system, human hippocampal neural stem cells (HIP-009, Vesta Biotherapeutics) were used as a sensitive biological model. These cells were cultured directly on a range of substrates, representing both scaffold fabrication materials and peripheral chip assembly components.

**Figure 12:**
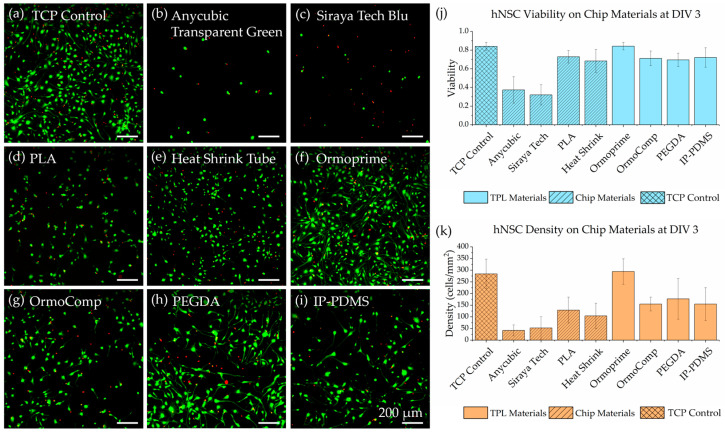
Viability assay of hNSCs at DIV 3 on a microfluidic chip and TPL materials. Live cells are marked green using calcein-AM, and dead cells are marked red using ethidium homodimer-1. Example 10× images of the assay are shown: (**a**) TCP control, 3D-printed needle holder materials, including (**b**) Anycubic Transparent Green LCD resin, (**c**) Siraya Tech Blu resin, (**d**) PLA polymer, (**e**) heat-shrink tubing, (**f**) Ormoprime, (**g**) OrmoComp, (**h**) PEGDA, and (**i**) IP-PDMS. (**j**) Average viability of hNSCs on each material. (**k**) Average live cell density of hNSCs on each material.

Representative fluorescence images from the Live/Dead viability assay, performed on day in vitro (DIV) 3, are shown in Figure 12a–i. The tested substrates were divided into two groups. The first group focused on the photopolymerizable resins used directly for scaffold fabrication, which are central to the microvascular chip system. These included Ormoprime adhesion promoter, OrmoComp organic–ceramic polymer, PEGDA 700 hydrogel, and IP-PDMS elastomer. These materials form the functional scaffold architecture, confirming their compatibility with hNSCs was particularly critical.

The second group included chip assembly materials such as tissue culture plastic (TCP) as a positive control, heat-shrink tubing, and three needle holder materials: polylactic acid (PLA) fabricated via fused deposition modeling (Creality Ender 3, Hebron, KY, USA), and two photopolymer resins (Siraya Tech Blu (San Gabriel, CA, USA) and Anycubic Transparent Green (Grace Place, CA, USA)) fabricated with an LCD 3D printer (Anycubic Photon Mono 4k, Grace Place, CA, USA). Notably, the LCD resin needle holder was placed on the tops of culture wells and did not directly contact the cell culture medium.

Quantitative viability analysis is summarized in Figure 12j, with live cell densities shown in Figure 12k. The results revealed suitable cytocompatibility exceeding 70% for OrmoComp, PEGDA 700, and IP-PDMS materials, which was also comparable to heat-shrink tubing and PLA. Moreover, these materials supported consistent hNSC adhesion and spreading, confirming their suitability for neural culture applications. TCP and Ormoprime-treated surfaces exhibited the highest viability at around 84%.

In contrast, the LCD resin needle holder demonstrated pronounced cytotoxicity. Both Siraya Tech Blu and Anycubic Transparent Green needle holders reduced cell viability to below 40%, and live cell density analysis revealed markedly lower cell attachment and spreading. These findings suggest that the release of volatile compounds from LCD materials negatively impacted nearby cells, even in the absence of direct media contact. These printing materials should be avoided as microfluidic chip materials in future studies.

## 4. Discussion

This work advances the design and fabrication of microfluidic chips with embedded microvasculature scaffolds using TPL, addressing key challenges in generating physiologically relevant vascular structures. Compared to conventional CAD workflows, the mesh-based modeling strategy implemented in Rhinoceros 3D provided an efficient and flexible approach for creating complex scaffold geometries suitable for biological applications. By enabling rapid design iteration and integration of user-defined pore architectures and supports, this workflow streamlined the fabrication of customized vascular networks.

A persistent challenge in TPL fabrication of microcapillary scaffolds is maintaining open-pore architectures, particularly along curved surfaces and scaffold edges. Due to the anisotropic voxel dimensions of TPL, pores facing lateral directions are especially prone to closure during printing. In this study, these limitations were addressed by elongating holes of the subsurfaces on the sides of the capillary membranes, effectively preserving side-facing pore openings and improving overall printing fidelity. Additionally, support strategies were critical for maintaining scaffold integrity throughout printing and post-processing. Polygonal support rings were sufficient for planar capillary designs but were inadequate for more complex geometries. Placement of lattice nodes at FOV stitching boundaries proved effective for reducing misalignment and ensuring seamless scaffold fabrication. In contrast, lattice supports, optimized for fully 3D scaffolds, provided distributed contact points that enhanced structural robustness and minimized deformation at the FOV intersections. The lattice supports were insufficient to provide structural stability for softer print materials such as PEGDA 700 and IP-PDMS. PEGDA 700 was shown to deform initially in an aqueous environment. PEGDA 700 has been shown to soften significantly in aqueous conditions, reducing from the measured modulus of 30.7 MPa down to a reported 0.5–5 MPa [35]. This softening is likely responsible for the initial geometry change and deformation. IP-PDMS also has a lower reported elastic modulus (350 kPa–17.5 MPa), ultimately leading to deformation if left unsupported [33]. In contrast, the mesoscale cone supports, printed in OrmoComp, offered robust structural reinforcement, enabling successful fabrication of 3D capillary architectures even with soft materials. Both PEGDA and IP-PDMS demonstrated strong adhesion to the OrmoComp base, establishing a rigid interface that effectively anchors the soft TPL-printed scaffolds.

The ability to realize complex 3D capillary geometries using additive fabrication offers distinct advantages over existing subtractive and planar fabrication approaches. Previous studies relying on planar TPL geometries or subtractive methods, such as ablating hydrogels to create channels, are inherently limited in spatial complexity and integration potential. In contrast, the additive approach demonstrated here enables freeform capillary architectures to be fabricated directly within microfluidic devices without reliance on sacrificial layers or extensive assembly. This capability opens opportunities for more advanced 3D cell culture models, as cells can migrate into and interact with the scaffold lattice, potentially guiding cellular organization and enhancing physiological relevance. Furthermore, by reducing the use of PDMS relative to conventional microfluidic chips, this system avoids small molecule leaching issues and creates a more biochemically compatible environment for sensitive biological assays.

A novel microfluidic interfacing strategy was also developed, utilizing heat-shrink tubing and hypodermic needles to produce reliable and low-cost connections between the external fluidic system and the printed scaffold. This approach eliminated the need for bulky adapters and enabled a compact chip design. SEM analysis confirmed that the heat-shrink tubing formed tight seals without intruding into the scaffold lattice regions, preserving perfusion pathways. Perfusion experiments, performed using a push–pull syringe pump and PIV analysis, validated the suitability of this system for future cell studies.

OrmoComp, PEGDA 700, and IP-PDMS all supported hNSC adhesion and viability, each exceeding 70% viability after 3 days in culture. Among the peripheral chip components, PLA lids demonstrated good compatibility, supporting healthy cell attachment and viability comparable to standard culture substrates. In contrast, LCD 3D-printed resins used for chip lids, despite not contacting the media directly, exhibited significant cytotoxicity, likely due to volatile compound release or insufficient post-curing. Although Siraya Tech Blu resin nominally meets ISO 10993-5 biocompatibility standards [36], the curing conditions applied in this study proved inadequate. These findings underscore that even non-contact components must be carefully evaluated to avoid adverse effects on sensitive neural cultures.

## 5. Conclusions

In this study, we developed a versatile and modular platform for fabricating perfusable microvascular scaffolds integrated within microfluidic chips using TPL. Leveraging mesh-based design, multi-material printing, and an accessible heat-shrink tubing interface, we successfully produced customizable 3D capillary networks with tunable mechanical properties.

Rigid (OrmoComp), moderately stiff (PEGDA 700), and soft elastomeric (IP-PDMS) materials were all successfully printed into a multi-material chip configuration. However, PEGDA 700 and IP-PDMS printed structures suffered from deformation after development, requiring a cone-based support structure to maintain fidelity. Flow validation demonstrated that the printed scaffolds supported perfusion at physiologically relevant flow speeds.

Cytocompatibility assays further confirmed that all TPL-printed scaffold materials supported robust human neural stem cell viability and attachment, validating their suitability for neurovascular and brain-on-chip applications. PLA lids demonstrated suitable biocompatibility, while LCD resin lids exhibited significant cytotoxicity, underscoring the importance of carefully selecting and processing all chip components to ensure compatibility with sensitive biological environments.

Together, these results establish a versatile and adaptable platform for creating biologically relevant 3D vascular scaffolds in microfluidic systems. Future efforts will focus on incorporating brain endothelial cells to create biomimetic BBB models and integrating microcapillary scaffolds into brain organoid systems to promote maturation and reduce necrosis. These advancements will expand the platform’s utility for neurovascular modeling, disease research, and drug transport studies.

## 6. Patents

Nathaniel Harris and Min Zou, “Customizable Vascularized Lattice for In-vitro Brain Models”, U.S. Provisional Patent Application No. 63/746,073, filed 16 January 2025.

## Data Availability

The data used in this study are available upon reasonable request from the corresponding author.

## Abbreviations

The following abbreviations are used in this manuscript:

TPL	Two-photon lithography
PDMS	Polydimethylsiloxane
PLA	Polylactic acid
LCD	Liquid crystal display
BBB	Blood-brain barrier
NVU	Neurovascular unit
PEGDA	Polyethylene glycol diacrylate
NURBS	Non-uniform rational b-spline
FOV	Field of view
CAD	Computer-aided design
DiLL	Dip-in laser lithography
bFGF	Basic fibroblast growth factor
EGF	Endothelial growth factor
UV	Ultraviolet
PBS	Phosphate-buffered saline
SEM	Scanning electron microscope
DIV	Days in vitro
